# *Plasmodium ovale* infection in Sri Lanka: distant exposure and incidental detection of hyperparasitemia: a case report

**DOI:** 10.1186/s13256-023-04226-z

**Published:** 2023-12-12

**Authors:** Damsara Kularatne, Pubudu Chulasiri, Arinda Dharmapala, Senanayake Kularatne

**Affiliations:** 1 Center for Research in Tropical Medicine, Faculty of Medicine, University of Peradeniya, Peradeniya, Sri Lanka; 2 Anti Malaria Campaign, Ministry of Health, Colombo, Sri Lanka; 3 Department of Surgery, Faculty of Medicine, University of Peradeniya, Peradeniya, Sri Lanka; 4 Department of Medicine, Faculty of Medicine, University of Sri Lanka, Colombo, Sri Lanka

**Keywords:** Case report, *Plasmodium ovale* malaria, Bacterial co-infection, Hyperparasitaemia, Anopheles mosquito, India, Sri Lanka

## Abstract

**Background:**

*Plasmodium ovale* malaria, which was previously endemic to tropical Africa and the Southwest Pacific islands is now being reported from parts of Asia. In Sri Lanka, the indigenous transmission of malaria has not been documented since October 2012. Since then, there have been several imported cases of malaria, including *P.* *ovale*, which have been detected sporadically. The reporting case of *P*. *ovale* was imported and detected incidentally in 2021, with several atypical presentations.

**Case presentation:**

A 40-year-old Sri Lankan medical doctor developed continuous fever with chills, rigors, and dysuria a day following removal of a large lipoma at the root of the neck under general anaesthesia. When the fever has been responding to antibiotics, on the 4th postoperative day a mild thrombocytopenia on complete blood count was detected. A blood smear which was done on the 5th postoperative day incidentally found a malaria parasite and confirmed as *Plasmodium ovale* with a density of 6535 parasites/microliter on the same day*.* He never had malaria in the past, but he had worked in South Sudan 1 year ago and visited India six months ago. On the 6th postoperative day, he was treated with chloroquine, and hyperparasitemia reduced rapidly by the next day. As the fever recurred with clinical deterioration, he was treated with different antibiotics. During the course of the illness, he did not develop pallor, or icterus except for a palpable soft spleen. The parasite count was zero on the 9th postoperative day and his fever subsided on the next day. Further, he was treated with primaquine to prevent future relapse and transmission.

**Conclusion:**

A long incubation period, incidental detection of *P ovale* in a blood smear, and hyperparasitaemia are the atypical presentations of this case. Postoperative bacterial infection and stress may have reactivated the dormant malaria (hyponozoites) in this patient with an unusual picture. Coinfection of malaria with bacterial sepsis is a challenge in the management of the patient. As the Anopheles mosquito vector exists in Sri Lanka, the risk of indigenous transmission is high from such imported cases of *P. ovale*.

## Background

Globally and nationally, malaria is an important tropical disease caused by Plasmodium species. Five Plasmodium parasite species are known to cause malaria in humans. They are *P. falciparum, P.* *vivax, P.* *malariae, P.* *ovale, and P.* *knowlesi.* Of them, falciparum and vivax are the dominant species of global malaria causing high mortality and morbidity rates. *Plasmodium ovale*, which was traditionally endemic to tropical Africa, New Guinea, eastern parts of Indonesia, and the Philippines [1, 2] is now being reported from parts of Asia including India, Indonesia, Laos, Myanmar, Thailand, Vietnam, and Cambodia [3–7]. Although *P.* *ovale* is usually associated with low morbidity and mortality, previous studies have reported several complications that can be fatal [8].

In Sri Lanka, the indigenous transmission of malaria has not been documented since October 2012, and thereafter the World Health Organization (WHO) declared malaria-free status in September 2016 [9]. The first published case of *Plasmodium ovale* was detected in Sri Lanka in 2003, and it is considered to be indigenous [10]. In 2017, the second published case of *Plasmodium ovale* infection in Sri Lanka was considered to be imported [11]. The Antimalaria Campaign (AMC) of Sri Lanka is the principal organization that sustains the elimination status of malaria in the island and monitors all newly detected cases and categorizes them accordingly as imported cases. According to its annual report, 17 imported cases of *P. ovale* have been detected in Sri Lanka from 2013 to 19 [12]. After 2019 these numbers have increased and total up to 58 according to the unpublished data belonging to AMC. We report an incidentally detected imported case of *P. ovale* in Sri Lanka highlighting the way of detection and its atypical presentation.

## Case presentation

A 40-year-old Sri Lankan medical doctor from Kandy, Sri Lanka developed continuous fever with chills, rigors, and dysuria without loin pain, a day after removal of a large benign subcutaneous lipoma at the root of the neck under general anesthesia in November 2021. He had to remain fasting without drinking water as a prerequisite for general anaesthesia for six hours. His fever was attributed to a postsurgical infection, possibly a urinary tract infection despite a lack of predisposing factors such as urinary bladder catheterization and co-morbidities such as diabetes mellitus. He had received Intravenous Co-amoxiclav for 5 days empirically with temporarily declining fever spikes. The complete blood count done on the 4th day after surgery showed a total white cell count of 5.3 × 10^9^/L, hemoglobin of 13 g/dl, and platelet count of 140 × 10^9^/L. The C-reactive protein (CRP) was 40 mg/L. Detection of mild thrombocytopenia on complete blood count had prompted to do a blood smear on the 5th postoperative day which found the malaria parasite incidentally. At this juncture, the anti-malaria campaign in Sri Lanka, was informed about the detection of malaria as a compulsory regulation. On the same day, the regional malaria officer of the anti-malarial campaign visited the patient and obtained blood for thick and thin blood smears and rapid detection tests for malaria. A trained microscopist of AMC identified diagnostic characteristics supporting *Plasmodium ovale*. Further, higher density of *Plasmodium ovale* (≥ 1000 parasites/µl) [1]was counted and calculated to be 6535 parasites/microlitre according to the blood smear, while the rapid antigen detection test (RDT) was negative for both vivax and falciparum. Furthermore, PCR (Polymerase Chain reaction) for *P. falciparum* was performed later, and it was ruled out. On the next day, the patient was started on treatment for malaria according to the National Guideline on Malaria Management 2014 [13]. He was treated with chloroquine 25 mg/kg body weight for 3 days for ovale malaria divided as 10 mg/kg on the first and second day followed by 5 mg/kg body weight on the third day, while the parasitic density was found to have increased to 7225 parasites/microliter. Although there was a rapid decline in parasitemia to 976 parasites/microliter within 24 h with chloroquine, he again developed a high temperature on the 7th post-operative day along with further reduction in platelet count from 140 to 112 × 10^9^/L Escalation of bacterial sepsis was considered, and he was treated with intravenous meropenem on the same day after taking blood for culture. However, due to clinical deterioration and financial constraints, he was transferred to a government hospital where meropenem was not available on that day. Then the antibiotic was changed to Piperacillin-Tazobactam on the 8th postoperative day. His urine and blood cultures gave negative growth probably due to prior empirical antibiotic treatment. His temperature declined and he felt better after 2 days of antibiotics which was continued for 5 more days until full recovery. Afterward, he was given 0.25 mg/Kg body weight per day of primaquine for 14 days with the intention of preventing transmission by gametocytes and preventing relapse by hypnozoites after excluding Glucose-6-phosphatase dehydrogenase (G6PD) deficiency by performing a G6PD assay as G6PD deficiency is prevalent in Sri Lanka [14].

On further inquiry, he never had malaria in the past, but he had worked in South Sudan, until December 2020 for 1 year. During that time, he had taken mefloquine prophylaxis properly according to the National guideline on malaria prophylaxis for travelers 2019 [15].Being a medical doctor he claimed that South Sudan is free of *ovale* infection but known to have *P.vivax* and *P. falciparum* transmission [16]. After returning to Sri Lanka, 3 months later in March 2021, he visited Delhi India for 5 days. During that time, he did not take prophylaxis due to a short visit and he was aware of the existence of *ovale* infection in India [17].Since then, he remained healthy and active until his recent surgical history. He was in good health in the past and he is married and has two children. He used to take a balanced diet without restrictions and haven’t had any food allergy. He has been working as a medical officer and denies any high-risk sexual behaviors and he has not been taking any long-term medication, blood transfusions, or intravenous drugs. He had an averagely built body and during the course of the illness, he did not develop anemia or icterus. His blood pressure was around 110/80 mmHg and the rest of the examination was normal except for the palpable soft spleen.

**Fig. 1:**
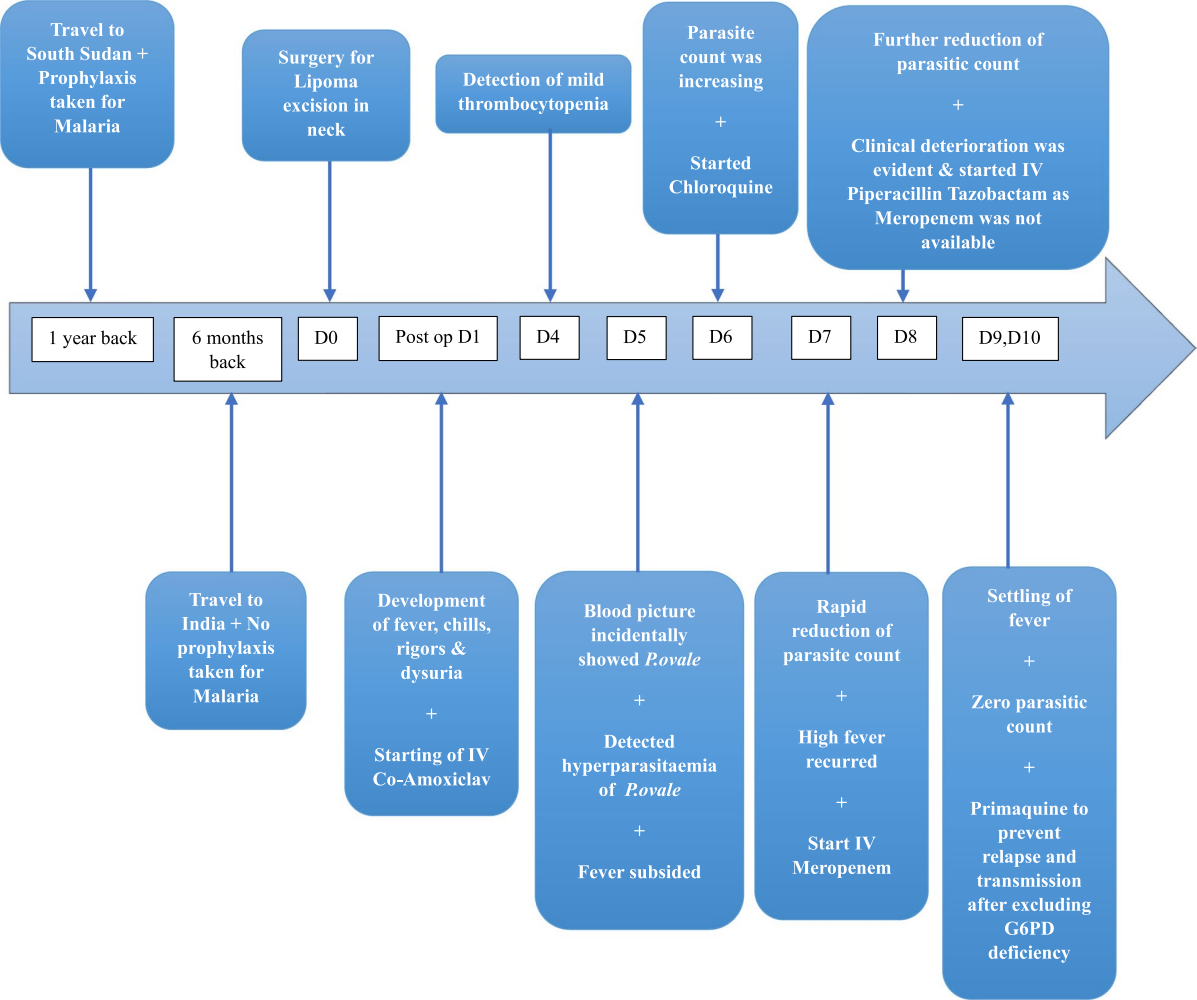
Important days and Presentations of the case as a Timeline

The investigations including hematological, and biochemical parameters, and parasitic counts during the illness are shown in Tables 1, 2, and 3. He underwent an ultrasound examination of the abdomen twice where the first scan on the 5th post-operative day showed mild heterogenic echotexture (Right Kidney 10.5 × 5 cm, Left Kidney 11.3 × 5.6 cm) with a mildly thickened bladder wall suggestive of mild pyelonephritis and cystitis. The second ultrasound scan on the 9^th^ post-operative day showed resolving features of pyelonephritis. He made a full recovery by the 10th postoperative day, remained asymptomatic, and later migrated to another country. The important days and presentations of the case are depicted as a timeline in the Fig. 1 below.

**Table 1:** Hematological and urinary investigations

November 2021 Test	Post operative day						Normal values
	4	7	8	9	10	11	
Complete blood count (× 10^9^/L)							
White blood cell count	5.3	5.8	4.89	4.3	3.74		4–11
Neu%	86.7	70.4	70.7	48.3	44		40–60
Lym%	7.6	19.8	19.3	28.9	42.2		20–40
Mono%	5.1	9.3	9.3	10.3	11.4		2–8
Eosi%	0.2	0.0	0.3	1.6	1.6		1–4
Hb (g/dL)	13.5	13.2	12.5	12.3	11.8		13.5–17.7
RBC	4720	4620	4620	4520	4360		4500–6500
MCV (fl)	83.9	82.3	84.1	84.1	84.1		80–96
HCT (%)	39.6	38.0	38.9	38	36.6		40–54
MCH (pg)	28.6	28.6	27.2	27.1	27.1		27–32
MCHC(g/dL)	34.1	34.7	37.1	32.2	32.2		32–36
RDW (%)	13.2	13.4	15.5	13.5	13.8		11.8–14.5
PLT	140	112	67	78	100		150–450
Reticulocyte count (%)	–	–	–	0.73	0.66		0.5–2.5
C-reactive protein (mg/L)	42.9	146.8	–	300.9	198.8	122.2	< 5
Serum Creatinine(mg/dl)	1.07	1.26	1.21	1.6	0.92	0.81	0.6–1.5
Serum Na^+^ (mEq/l)	–	–	–	136	135	138	135–146
Serum K^+^ (mEq/l)	–	–	–	3.56	3.72	4.04	3.5–5.0
Urea (mg/dl)	27.8	–	–	–	–	–	8–25
Blood urea nitrogen (mg/dl)	13.1	–	–	–	–	–	6–24
Urine full report							
Protein	Nil	Trace	–	–	–	–	Nil
Glucose	Nil	Nil	–	–	–	–	Nil
Bilirubin	Nil	Nil	–	–	–	–	Nil
Pus cells/HPF	Occ	2–4	–	–	–	–	< 4
Red cells/HPF	1–2	12–20					< 4

**Table 2:** Biochemical investigations

November 2021 Test	Post operative day			Normal values
	4	10	11	
Liver profile (serum)				
Serum protein (g/l)	66.2	–	–	60–83
Albumin (g/l)	43.7	–	–	34–54
Globulin (g/l)	22.5	–	–	20–35
Bilirubin (mg/dl)	1.12	0.91	0.56	0.3–1.5
ALP (U/L)	91.8	–	–	39–117
ALT(U/L)	20.3	–	28.8	< 40
AST(U/L)	22.2	–	22.6	12–40
GGT(U/L)	10.9	–		10.5–58

ALP: Alkaline phosphatase; ALT: Alanine transaminase; AST: Aspartate transaminase; GGT: Gamma-glutamyl Transferase

**Table 3:** Daily Malaria parasitic count

Date	Post operative day				
	5	6	7	8	9
Density (/µl)	6535	7225	976	16	Negative
Species	*P. ovale*	*P. ovale*	*P. ovale*	*P. ovale*	–
Stages	T,G	T,G	G	G	–

T – Trophozoites G- Gametocytes

## Discussion

We report incidental detection of *ovale* malaria in a postsurgical patient who developed bacterial sepsis probably due to a urinary tract infection. His travel history suggests that his exposure to malaria would have happened 6 months to 1 year back either in India or South Sudan. Since then, he remained healthy until his recent surgical history. However, along with the postsurgical infection, he has developed hyperparasitemia as detected on the 5th and 6th days after surgery and its contribution to the ongoing fever is not known. His *ovale* malaria parasitemia cleared quickly with chloroquine but overall sepsis required a strong antibiotic course. He did not have susceptible factors for pyelonephritis, which probably led to sepsis, such as urinary bladder catheterization and immunocompromised states, except for not drinking water for six hours as a prerequisite for general anaesthesia. There are some questions that need inquiry based on this case. They are the asymptomatic long latent phase of the infection, then hyperparasitemia probably augmented by postsurgical stress and infection, and the doubtful contribution of malaria infection to the worsening of sepsis. Additionally, the absence of significant hemolysis was observed despite hyperparasitemia and rapid response to chloroquine suggests the effectiveness of the drug in treating ovale malaria in the background of chloroquine resistance in other species of malaria.

*Plasmodium ovale* was first described by a publication in 1922 by J.W.W Stephens by observing the blood of an East African patient [18]. Recent molecular methods have demonstrated that *P.ovale* consists of two essential subspecies namely *P.ovale curtisi* and *P.ovale wallikeri.* The finding was based on analyses of nucleotide deletions and substitutions in the 18S rRNA gene. These two types of *P.ovale* were shown to have distinct sequences for ookinete surface proteins [1, 19].

*Plasmodium ovale* accounts for 0.5% to 10.5% of all malaria cases globally. But, similarities with P.vivax such as similar morphology, ability to relapse, and tertian periodicity give the likelihood of misidentification, with the possibility of underestimating the prevalence of *P.* *ovale* [20]. However, only one study reported deaths due to *P.* *ovale* infection. The mortality rate of severe *P.* *ovale* infection in that study was 0.22% [21].

*Plasmodium ovale* is transmitted by the bites of infected female Anopheles mosquitoes. The only natural host of *P.* *ovale* is humans. Therefore, the developmental cycles of *P.* *ovale* can be identified in humans and vector mosquitoes. In humans, sporozoites from mosquito bites, rapidly invade the liver, and eventually, two forms are produced. They are merozoites, which invade reticulocytes and initiate the erythrocytic cycle, and hypnozoites which are dormant parasites that remain suspended in the liver. As *P.* *ovale* development is restricted to younger erythrocytes, maximum parasitic counts are usually low compared to *P. falciparum* and *P.* *vivax* infection [1]. In our patient, hyperparasitaemia can be considered an unusual failure of various host and parasite control mechanisms such as nonspecific host defense mechanisms, splenic clearance function, schizont development, and level of exhaustion of susceptible erythrocytes [22]. In that regard, food has a very significant role in suppressing illness specially those where host cell physiology has a direct role in determining the course of illness, particularly by involving molecular mechanisms driving epigenetically [23].

The symptomatic infection shows a tertian pattern of fever (every 48 h) with a feeling of wellness in between fever episodes. Hyperpyrexia accompanied by rigor, profuse sweating with defervescence of fever and gradual recovery over 6–12 h can be seen in typical infection. In the course of illness anemia and splenomegaly may develop. Usually in the natural course of the infection, the illness gradually resolves within a few weeks. On the contrary, our patient had a long latent period after exposure, and the clinical illness was superseded by postsurgical sepsis [24] Bacterial coinfections with malaria is known to exist that could change the clinical picture, complications and outcome of the patient. In a systematic review of 51 studies involving 1583 cases done in 2022, has found a pooled prevalence of deaths among patients with coinfection as 15%. Therefore, detection and treatment of coinfection is important [25].

Previous studies have reported several complications associated with *P.ovale*, despite its low morbidity and mortality such as Acute Respiratory Distress Syndrome (ARDS), renal impairment, hyperbilirubineamia, and hypotension [8].

The mean incubation period of primary infection by *P.* *ovale* malaria is approximately 2 weeks. The longest incubation period observed in a recent study was 265.71 weeks [26]. As mentioned above, hypnozoites, which are dormant stages in the liver can cause disease relapse in weeks, months, or years after the initial infection [27]. A relapse typically occurs when a patient, who had previously been infected and treated for a particular condition, experiences a recurrence of that condition. In this case of this patient, he has neither been infected by malaria nor undergone any antimalarial treatment for definitive malaria infection. Therefore, it would be more accurate to describe this condition as a reactivation of dormant malaria parasite rather than a relapse. Hence, in our patient, reactivation of hypnozoites is likely to be the cause of infection rather than a primary infection, as the incubation period of the index case is more than the mean incubation period of the primary infection. There is hardly any literature on the possible causes of the reactivation of hypnozoites. Reactivation might have been triggered by postsurgical stress and urosepsis. Primary infection with a long incubation period is supported by a phenomenon that is mainly attributed to variations in parasitic lineages and the use of chemoprophylaxis [16].

The gold standard of Malaria diagnosis is microscopy of thick and thin blood smears preferably stained by Giemsa stain. While thick blood smears are used to detect the presence of the malaria parasite, thin blood smear is used to identify the species. Though the microscopical appearance of *P*. *vivax* and *P*. *ovale* is closely similar, still differentiation is possible due to characteristic features in parasitic stages found in the blood. Molecular diagnostic methods like PCR and RDT need to be carried out for further confirmation of differentiation [28].

Treatment of *P.* *ovale* infection consists of treating the erythrocytic forms. Erythrocytic forms of *P.* *ovale* infection are highly susceptible to chloroquine. Hypnozoites that contribute to a relapse of the infection are eradicated by primaquine other than prevention of transmission by gametocytes. Primaquine should be started after the subsiding of fever after confirmation of normal glucose-6-phosphate dehydrogenase status [27]. It is unknown whether *P.ovale* has developed resistance to any antimalarial drugs to date [29]. Accordingly, our patient responded well to chloroquine with a rapid reduction in hyperparasitemia, and then he was treated with primaquine to prevent relapse and transmission.

Considering the prevalence of *P.* *ovale* infection in South Sudan where the index patient had worked, the literature reports that *P.* *ovale* is extremely rare [16]. In Delhi, India where he traveled next, *P.* *ovale* has presented as mixed infections (0.3%) along with other plasmodium species, such as vivax and falciparum [17, 30].

In Sri Lanka, the first case of *P.* *ovale* infection was detected in a 37-year-old male from Northwestern Province in 2003. He had neither a travel history overseas nor blood transfusions and it was considered as an indigenous case because during that time indigenous transmission of other malaria species was occurring in Sri Lanka. This isolated case raises the possibility of zoonotic *P.simiovale* infection because the DNA primers have not been evaluated against the DNA of the closely related Simian Parasite [10]. Infection with *P. simiovale* is prevalent in Macaca sinica monkeys in Sri Lanka [31]. The question of the existence of dormant forms of ovale malaria in humans is a possibility, but there has been no research carried out so far in Sri Lanka.

## Conclusion

Interestingly, the index case of ovale in Sri Lanka was detected incidentally and managed meticulously with the guidance of the Antimalaria Campaign of Sri Lanka. Coinfection of malaria with bacterial sepsis is a challenge in the management of the patient. Even though the 1st case claimed to be indigenous in origin, the index case has a strong travel history to both Sudan and India. It is important that Sri Lanka should be vigilant about imported cases of *P.ovale* as it might initiate indigenous transmission due to the presence of the Anopheles mosquito in the island. Findings such as hyperparasitaemia of *P*. *ovale* infection and prolonged incubation time with incidental detection need further studies to understand unknown dimensions of the pathogen. Postoperative infection and stress may have surfaced malaria in this patient with an unusual picture.

## Data Availability

Available in the process of authors.

## Abbreviations

AMC: Anti Malarial Campaign
ARDS: Acute Respiratory Distress Syndrome
CRP: C-Reactive Protein
G6PD: Glucose-6-phosphate dehydrogenase
HPF: High Power Field
PCR: Polymerase Chain reaction
Post Op: Post Operative
RDT: Rapid diagnostic test
WHO: World Health Organization
